# Ultrasound-Assisted Solvent Extraction of a Porous Membrane Packed Sample for the Determination of Tobacco-Specific Nitrosamines in the Replacement Liquids for E-Cigarettes

**DOI:** 10.3390/molecules24244618

**Published:** 2019-12-17

**Authors:** Paweł Kubica

**Affiliations:** Department of Analytical Chemistry, Faculty of Chemistry, Gdańsk University of Technology, 11/12 Narutowicza Str., 80-233 Gdańsk, Poland; pawkubic@pg.edu.pl

**Keywords:** e-cigarettes, replacement liquids, tobacco-specific nitrosamines, porous membrane, liquid chromatography-tandem mass spectrometry

## Abstract

The content of tobacco-specific nitrosamines (TSNAs) possessing carcinogenic properties has been an important area of research since replacement liquids were introduced for e-cigarettes. A method for determining *N*′-nitrosonornicotine (NNN), 4-(methylnitrosamino)-1-(3-pyridyl)-1-butanone (NNK), *N*′-nitrosoanatabine (NAT), and *N*′-nitrosoanabasine (NAB) in replacement liquids for electronic cigarettes was developed using liquid chromatography–tandem mass spectrometry with electrospray ionisation (HPLC-ESI-MS/MS) in the multiple reaction monitoring mode. The sample preparation of replacement liquids was accomplished via the ultrasound-assisted solvent extraction of a porous membrane packed sample. The sample preparation proved to be successful in extracting the analytes, with recoveries from 87% to 105%, with coefficients of variation < 4.9%. Moreover, the linearity and limits of detection and quantitation (LOD, LOQ), together with repeatability and accuracy, were determined for the developed method. The proposed sample preparation and developed chromatographic method were successfully applied to the determination of TSNAs in 9 replacement liquid samples. The NNK and NNN were found to be most frequently detected (89 and 67%, respectively), with concentration ranges from 1.2–54.3 ng/mL and 4.1–30.2 ng/mL, respectively, while NAT was detected with frequency of 22% with range 1.7–2.5 ng/mL and NAB were found to be below the LOD in all samples.

## 1. Introduction

Electronic cigarettes, most commonly known as e-cigarettes, are becoming more and more popular as an alternative to traditional smoking and are intended to simulate or imitate the sensations of smoking tobacco [1]. In general, these devices are made of a battery, a mouthpiece, (in many cases) an advanced electronic control system, a tank with a heater to hold a liquid (known as e-liquid) containing nicotine, propylene glycol (PG), vegetable glycerine (VG) and flavouring additives [2]. Initially, such devices were sold to be helpful in smoking and nicotine addiction therapy. However, even liquids marked as nicotine-free contain up to 0.3 mg/g of nicotine liquid [3]. Many controversies have since arisen, and more studies are becoming focused on comparing the toxicity of e-liquids and their corresponding aerosols to traditional tobacco and tobacco smoke. Among many others, tobacco-specific nitrosamines (TSNAs) are a particular area of interest [4,5], because they are present alongside nicotine and are known for their cancerogenic properties [6].

The nicotine used for the production of e-liquids is of natural origin and extracted from tobacco leaves. Hence, it was proven that, in many cases, corresponding TSNAs are also present [7,8,9,10]. The four most common TSNAs are: *N*′-nitrosonornicotine (NNN), 4-(methylnitrosamino)-1-(3-pyridyl)-1-butanone (NNK), *N*′-nitrosoanatabine (NAT), and *N*′-nitrosoanabasine (NAB). NNN and NNK are known to have cancerogenic effects on humans and are classified as Group 1 by the International Agency for Research on Cancer, while NAB and NAT are suspected to be cancerogenic and not cancerogenic, respectively [6,9,11]. These compounds are formed from alkaloid precursors together with the active participation of nitrites and nitrates. NNN, NAB, and NAT are mainly formed from their corresponding secondary amines (nornicotine, anabazine and anatabine, respectively) in the early stages of tobacco drying and processing, while NNK is produced in nicotine during the later stages of tobacco curing and fermentation [12]. As mentioned before, TSNAs can be extracted together with nicotine from tobacco leaves. Liquid and aerosol TSNAs occur at trace levels (up to several dozen ng/mL of e-liquids or puffs) or at the limits of the detection or quantitation of the analytical procedure used [4,13]. However, some research indicates increased levels of these compounds in e-liquid samples, when sample preparation is based on solid phase extraction (SPE) or liquid–liquid extraction (LLE) [9].

Presently, the technique of choice for the determination of TSNAs is liquid chromatography coupled with mass spectrometry (LC-MS) or tandem mass spectrometry (LC-MS/MS) [14,15,16,17,18,19,20,21]. However, gas chromatography coupled with a thermal energy analyzer (GC-TEA) was successfully applied to determine these analytes in tobacco and tobacco smoke [22,23,24,25], including analysis of other tobacco-related products [26]. Several papers are focused on determining TSNAs in e-liquids and aerosols using LC-MS/MS [9,13,27,28]. The analytes in e-liquids and in corresponding aerosols are at trace levels; hence, dilution of the sample and the presence of a matrix may strongly influence the detection and reliability of the obtained results.

This paper presents a novel method for the determination of TSNAs using the ultrasound-assisted solvent extraction of porous membrane packed sample followed by LC-MS/MS. The main idea of this method is to place e-liquid into a bag made of a polypropylene (PP) porous membrane and perform ultrasound-assisted extraction with an organic solvent. Originally, this technique used a solid sorbent inside the prepared bag immersed in the liquid matrix [29]. This approach was later modified for solid samples and introduced by Sajid et al. [30] and for liquids samples by Robles et al. [31] as an alternative to LLE and SPE.

For the first-time (according to the author’s knowledge), in the proposed method for extracting TSNAs, a liquid sample was placed inside a bag made of a PP porous membrane followed by the determination of TSNAs with LC-MS/MS. The presented approach could be treated as a green alternative for the determination of TSNAs by SPE and LLE techniques, due to its time reduction, lower generation of waste, solvent use, and the low cost of sample preparation.

## 2. Results

### 2.1. Detection Parameters and Chromatographic Separation

In order to determine the TSNAs, the MS/MS parameters and the ion source were optimized. For this purpose, separate solutions of the analytes NNK, NNN, NAT, and NAB with their deuterated internal standards (IS) NNK-d4, NNN-d4, NAT-d4, and NAB-d4 were prepared in H_2_O/ACN (40/60 *v*/*v*) to obtain 100 ng/mL of each. The mobile phase for the optimization of the multiple reaction monitoring mode (MRM) consisted of water and acetonitrile (ACN), and the chromatograph was set to work in the flow injection analysis mode (FIA) with a flow rate of 0.3 mL/min, while the injection volume was kept at 1 µL. For every compound precursor, an ion was chosen; in all cases, the most intensive ion was produced by protonation [M + H]^+^ in the positive mode. Each precursor ion was fragmented in the collision cell, and two specific (and most intense) product ions were chosen to construct the MRM transitions for each analyte and for its corresponding IS. In the next step, all voltages and m/z values were optimized automatically by the LabSolution software. Finally, all further chromatographic analyses were performed in the MRM mode. After optimizing the chromatographic parameters, the optimization of transitions and ion source parameters were repeated to increase the sensitivity for the chosen mobile phase and flow rate. The ion source parameters were chosen as follows: nebulizing gas flow: 3 L/min, heating gas flow and drying gas flow: 10 L/min, while interface, desolvation line and heat block temperature were set at 300, 250 and 450 °C, respectively. The capillary voltage was at 4kV. The parameters of the MS/MS are presented in Table 1.

**Figure 1:**
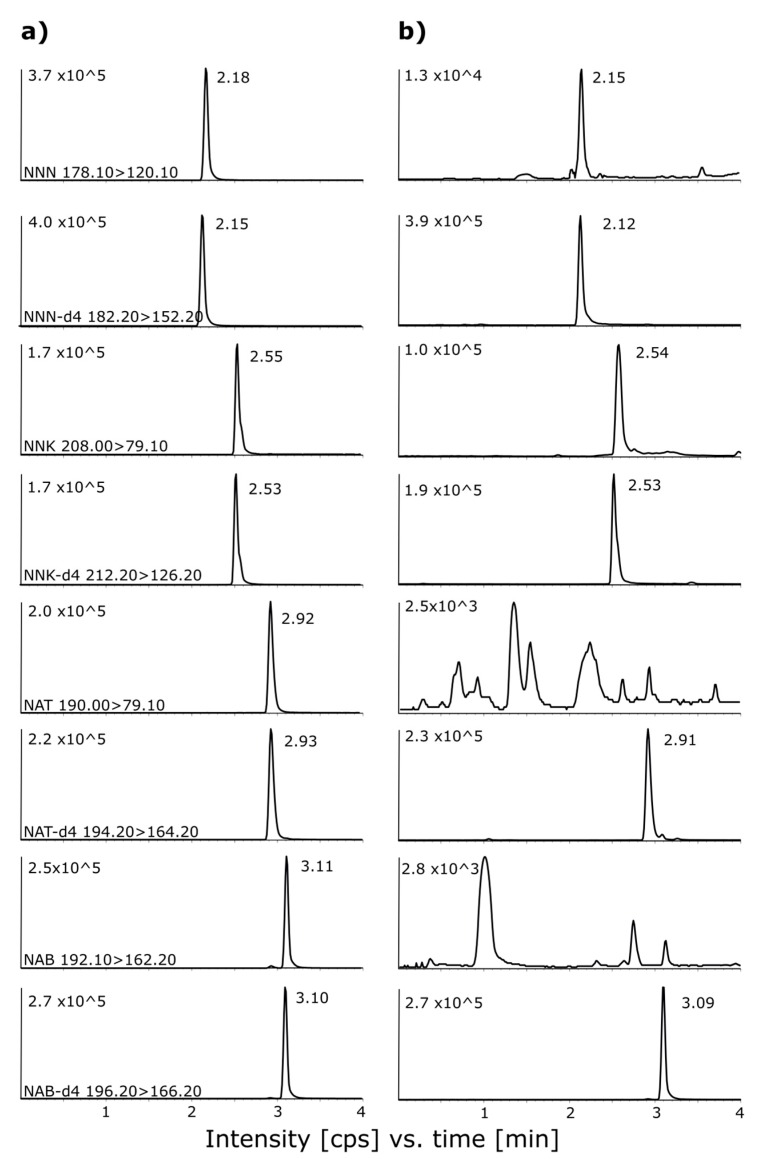
Chromatograms of (**a**) the spiked sample of the replacement liquid consisting of PG/VG 60/40 *v*/*v* with 20 ng/g of each analyte and 20 ng/g of each IS, (**b**) chromatograms of the real sample no 6.

In this study, a series of experiments was planned and performed to obtain separation of all four analytes with narrow peaks, a short analysis time (under 5 min), and good sensitivity. For all the separations, two columns were tested: Kinetex C18 100 × 2.1 mm, 2.6 µm and Kinetex C8 100 × 2.1 mm, 2.6 µm, both with a sorbent in core-shell technology, which improved the peak shapes and shortened the retention time. Different mobiles phases were tested, such as the organic component (B); methanol (MeOH) and ACN were chosen as aqueous component (A) buffers of ammonium formate and ammonium acetate with different pH ranges (3.5–8.5) and concentrations (5–30 mM). All separations were done in the gradient mode, which was optimized simultaneously together with the temperature of separation. The MeOH, as an organic component of the mobile phase, was eliminated due to the fact that the obtained retention times were longer and their peak shapes deteriorated (tailing factor > 1.4) compared to ACN (tailing factor 0.97–1.13). Regardless of the chosen chromatographic conditions, the coelution of NAT and NAB with resolution (R_s_ < 1) was observed for the Kinetex C8 column. A buffer pH below 6.7 significantly deteriorated the peak shape, due to the fact that the pKa values of the TSNAs range from 2.9 to 4.8. In this case, any tested buffer with a pH lower than 7 or water with formic or acetic acid addition falls into this range, where TSNAs are in an ionic form, so the peak deterioration/split is observed [9]. Moreover, a buffer content between 15 mM and 25 mM improved the peak shape, and further increasing the buffer content (30 mM) resulted in no significant changes. A comparison of ammonium acetate and ammonium formate buffers favors the second one due to the decreased width of the peaks at 5% of height from 0.14, 0.16, 0.14, and 0.15 min to 0.12, 0.13, 0.12, and 0.14 min for NNN, NNK, NAT, and NAB, respectively. The addition of acetone (ACTN) into the organic component of the mobile phase (ACN/ACTN 95/5 *v*/*v*) increased the R_s_ value (NAT-NAB) to 1.4. The chromatogram of comparison of ACTN influence is presented in the Supplementary Material, Figure S1. An increased temperature (50 °C) and a flow rate of 1 mL/min resulted in shortening the retention times for all analytes. Finally, the optimized method utilized a Kinetex C18 column, with a flow rate of 1 mL/min (A: 20 mM of ammonium formate buffer pH = 8.5, B: ACN/ACTN 95/5 *v*/*v*, gradient 0–1 min 5% B, 1–4 min 35% B), while the equilibration of the column was kept for 4 min at 5% of B, with a temperature of separation of 50 °C. The chromatograms of the spiked sample together with the chromatograms of the real sample are presented in Figure 1.

### 2.2. Optimization of the Extraction Procedure

**Figure 2:**
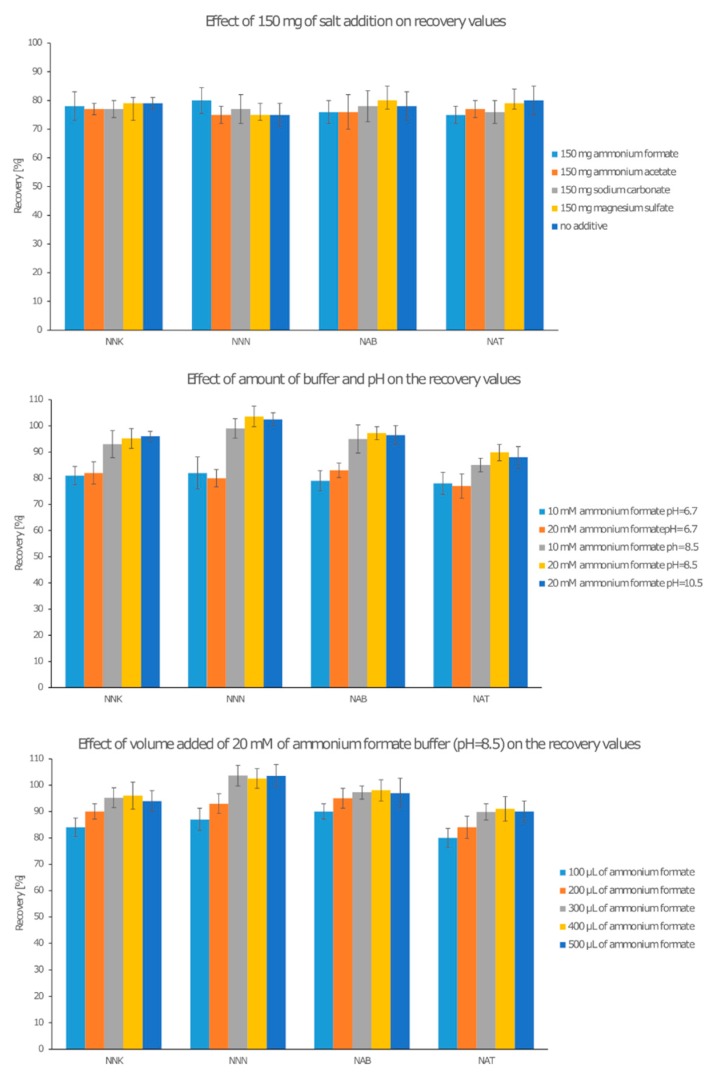
Chosen recovery values of tobacco-specific nitrosamines (TSNAs) for addition of salts and buffer during sample preparation step, (**a**) 150 mg of different salt addition including no addition, (**b**) amount of buffer used, (**c**) volume of 20 mM, pH = 8.5 ammonium formate buffer added.

The replacement liquids are mostly made of PG and VG. At present, the VG content is increased due to the fact that this substance, during aerosolization, is responsible for denser and more intense smoke. The laboratory produced replacement liquid consisting of PG/VG 60/40 *v*/*v* that was further spiked with TSNAs at 20 ng/g, and the content of IS was kept at 20 ng/g. Both main components are well miscible with popular solvents such as acetone, acetonitrile, water, and chloroform, so they were removed from the studies due to the possibility of diffusion through the membrane of PG and VG into the extraction solvent. To optimize the extraction procedure, two solvents were chosen: ethyl acetate and methylene chloride. However, during optimization, the second solvent was responsible for damaging the PP bags. All optimizations were done with ethyl acetate as an extraction solvent. Sheets of the PP membrane (200 × 200 mm, 0.1 µm pore diameter) were cut, folded, and sealed with a welder to construct an open bag. Then, 1g of the spiked liquid was placed inside the bag, together with one of the chosen additives (salts: ammonium formate, ammonium acetate, sodium carbonate, or magnesium sulfate (50, 100, 150, 200, 250 mg)) or a buffer of ammonium formate (10, 20, 40 mM, 100, 200, 300, 400, or 500 µL) with different pH values of 6.7, 8.5, or 10. Next, the bag was sealed and placed in a 15 mL test-tube with 6, 8, 10, or 12 mL of ethyl acetate and sonicated for 5, 10, 15, 20, or 25 min. After sonication, the bag was removed, and the solvent was evaporated under a stream of nitrogen. The residue was dissolved in 200 µL of the mobile phase 95/5 of ACN/ACTN (95/5 *v*/*v*)/ammonium formate buffer 20 mM pH = 8.5 and placed in an autosampler vial. The spiked samples without any additives or with a salt additive resulted in recoveries of 75–80% for all TSNAs; no significant changes in the recovery values were observed, regardless of the salt or amount. For the ammonium formate buffer with a pH above 6.7, a significant increase of the recovery values was observed for all analytes (90–105%). Further increasing the pH (above 8.5) resulted in no observable changes. A similar result was found for the amount of buffer. A slight increase (2–4%) was noted for 20 mM and 40 mM of buffer in comparison with 10 mM. No observable increase in recoveries were noted for a buffer amount above 300 µL inside the PP bag. Comparative figure of recoveries of TSNAs of chosen conditions were presented on the Figure 2. Similar to the chromatographic conditions, the pH values in the alkaline range were greater than the pKa values for all TSNAs (4.8 for the pyridine ring), thereby forcing the analytes to be present in non-ionic form. No significant changes were noted for the different volumes of ethyl acetate as the extraction solvent (6–12 mL). Hence, it was decided to use 6 mL as the minimum required to immerse the entire bag in the solution. The addition of a buffer into the spiked sample in the bag required no previous mixing, due to the subsequent sonication. Considering the time of sonication, an increase of the recoveries was observed up to 15 min, while for 20 and 25 min, no observable recovery increase was noted. The whole procedure of sample preparation, including the time of sonication, was no longer than 25 min. A detailed description of the sample preparation and the spiked samples for recovery estimation are presented in Section 4.3.

### 2.3. Method Validation

To evaluate analytical performance of the chromatographic method and the sample preparation procedure, the spiked samples were prepared with TSNAs of 5, 20, and 40 ng/g of replacement liquids, while the IS concentration was kept at 20 ng/g. The following parameters were studied: linearity, standard deviation of slope (S_a_), standard deviation of the constant term (S_b_), and the values for the limit of detection (LOD), which were based on formula LOD = 3.3S_b_/a [32] where *a* is the slope of calibration curve; the values for the limit of quantitation (LOQ) were calculated as 3 × LOD, and the coefficients of determination were R^2^. Further recoveries, in terms of precision, were evaluated for three spiking levels, each with *n* = 6 for the independent analysis and accuracy in terms of the coefficients of variation (CV). One series of the fortified sample (20 ng/g, *n* = 6) was analysed for the next three days in order to determine repeatability. All samples were prepared according to the protocol described in Section 4.3. Six calibration solutions with a mixture of analytes were prepared in the mobile phase initial conditions, as follows: 1, 5, 10, 20, 50, 75 ng/g. The IS concentration was kept at 20 ng/g. To increase the precision at lower ranges of the calibration curves, a weighing factor of 1/C was applied. All calibration solutions were prepared by weighing to avoid recalculation of the obtained results according to the density of the samples. Blank (with IS) and double-blank samples (without IS) were prepared, as well. No significant or intense peaks were noted for any prepared blanks close to the expected retention times of the analytes. The results of validation are presented in Table 2 and Table 3.

All constructed calibration curves were linear in the given concentration range, with R^2^ above 0.993 and LOD values below 1 ng/g. Calculation of the LOD based on the calibration curve equation is recommended due to the strict connection between the developed method and the obtained results. The highest recoveries (above 93%) were obtained for NNK, NNN, and NAB, while the NAT recoveries did not exceed 92%. The obtained results are satisfactory in terms of their precision, accuracy, and repeatability. Thus, the developed method was proven to be suitable for determining the trace amounts of TSNAs in the real samples.

### 2.4. Analysis of Real Samples

The nine samples were bought together at a local market from four producers. The nicotine content was 6 mg/mL in each sample (declared by the label of the producer), while the flavors of the replacement liquids were chosen according to their popularity. Samples were prepared by the developed protocol and analyzed by HPLC-MS/MS, *n* = 3. All results were recalculated to express the determined concentration in mg/mL of replacement liquids, knowing its density and mass taken. Results of the analysis are presented in Table 4.

The analysed samples came from four different producers. In each sample, at least one TSNA was present. The most commonly occurring analytes were NNK and NNN with detection frequency at 89 and 67%, respectively, while NAT was present only in two samples (22%), and NAB was not found in any sample (<LOD).

## 3. Discussion

The developed LC–MS/MS method proved to be suitable for the separation and determination of analytes in under 4 min. In most cases, NNN and NNK were detected as the two major TSNAs present in the replacement liquid samples. The reasons for the frequent presence of NNK and NNN are likely the storage, aging, and manufacturing process of the replacement liquids. However, the results in the literature on the determination of TSNAs are somewhat inconclusive; some groups report a high content of TSNAs in replacement liquids [9], which corresponds to this research where NNK and NNN were detected with high frequency 89 and 65% respectively. In other studies, contents of analytes are below the LOD or close to it [4,10,27], where Farsalinos et al. [4] detected mostly NAB at around 2 ng/mL and NNN at 7 ng/mL, while Goniewicz et al. [27] focused only on NNK and NNN in aerosol generated where levels of detected were up to 28.3 ng/150 puff and up to 4.3 ng/150 puffs. However, there is no comparison in this research between the content of TSNAs in replacement liquids and corresponding aerosols, hence no mass change tracking approach was implemented. There are few possible reasons for this discrepancy—the different content of nicotine in the replacement liquids could yield different ratios of detected TSNAs, and the simple dilute-and-shoot approach does not significantly reduce the matrix influence or enrichment factor of the sample preparation. Example chromatograms obtained after the dilution (100 × with mobile phase) of sample 6 and 9 are presented on the Figure S2. Moreover, the multistep extraction presented in a paper by Kim et al. [9] is omitted, the samples are not diluted, and the matrix influence is significantly reduced [4,13]. The ability of the main components (PG and VG) to interfere with the determination of TSNAs is strongly reduced, so high recovery values were obtained. It was proven that sample preparation based on this protocol was successful in extracting the analytes from replacement liquids for e-cigarettes. In terms of green chemistry, the proposed method uses less solvent (approximately 6 mL), produces less waste, saves time, and is easy to use and not as laborious as SPE or LLE.

Future research should focus on the determination of TSNAs at a larger scale to study the contents of these analytes across different producers and different amounts of nicotine in replacement liquids. According to the literature, the composition of replacement liquids reflects the composition of the produced aerosol [1]. Thus, it can be assumed that TSNAs could also be present in the formed aerosol. Moreover, the influence of heating on the occurrence of such analytes would be an interesting research avenue. The author believes that the proposed procedure may be successfully applied to the extraction of other compounds in replacement liquids (nicotine and their derivatives), successfully reducing the sample matrix influence and improving the reliability of the results.

## 4. Materials and Methods

### 4.1. Standards and Solutions Preparations

All samples of the four TSNAs (*N*′-nitrosonornicotine, 4-(Methylnitrosamino)-1-(3-pyridyl)-1-butanone, *N*′-nitrosoanatabine, and *N*′-nitrosoanabasine) were purchased from Merck (Darmstadt, Germany), while the deuterated samples (NNK-d4, NNN-d4, NAT-d4, and NAB-d4) were obtained from Toronto Research Chemicals (Toronto, Canada). All solvents used were of analytical grade (ACN, MeOH, acetic and formic acids, ammonia, ethyl acetate, and methylene chloride) and were purchased from Merck (Darmstadt, Germany). The sheets of PP membrane (200 × 200mm, 0.1 µm) were purchased from GVS Filter Technology (Lancaster, United Kingdom). Ultrapure water was prepared by an HLP5 system from Hydrolab (Wiślina, Poland), while PG (99.8%) and VG (99.5%) were obtained from Anwit (Warsaw, Poland). Individual stock solutions of analytes and their corresponding IS used in this study were prepared by dissolving appropriate amounts in the ACN solvent to obtain 40 µg/mL of each. Working standard mixtures were obtained by dilution in the mobile phase. Calibration solutions were prepared by adding the appropriate amount to obtain 1, 5, 10, 20, 50, and 75 ng/g of each analyte, while the IS concentration was kept at 20 ng/g of each. All solutions were stored in a fridge at 4 °C for up to 4 weeks.

### 4.2. Instruments and Analytical Conditions

The chromatographic system consisted of a UPLC system (Shimadzu, Japan) equipped with a DGU-20A5R degasser, a CBM-20A controller, two Nexera X2 LC-30 CE pumps, a X2 SIL-30AC autosampler and a CTO 20AC column oven. All analyses were done on a Shimadzu LCMS 8060 MS/MS in MRM mode with positive ionization, and the working parameters of the ion source and transition are presented in Table 1. All modules were controlled by LabSolutions, and this software was used for data collection and processing. The final optimized method for separation was using Kinetex C18 100 × 2.1 mm, 2.6 µm from Phenomenex (Torrance, CA, USA). The chromatographic conditions were as follows: a flow rate at 1 mL/min, component A of the mobile phase: 20 mM of ammonium formate buffer pH = 8.5; component B of the mobile phase: ACN/ACTN 95/5 *v*/*v*, gradient 0–1 min 5% B, 1–4 min 35% B; the equilibrium of the column was maintained for 4 min at 5% of B. The temperature of separation was set at 50 °C, while the injection volume was kept at 1 µL. The ultrasonic bath used for sample preparation was made by Bandelin Sonorex (Berlin, Germany) with ultrasonic peak power of 640 W with oscillating systems (nominal power 160 W), while ultrasonic frequency was at 35 kHz.

### 4.3. Sample Preparation of the Replacement Liquids and Spiked Samples

The model liquid consisted of PG and VG at 60/40 *v*/*v* and was prepared in the laboratory. A proper amount of TSNAs together with IS was added to the laboratory-made replacement liquid to obtain 5, 20, and 40 ng/g, while the IS concentration was 20 ng/g. Both the real sample and the spiked sample were prepared as follows: The exact amount (around 1g) was placed in the prepared PP membrane bag together with 300 µL of ammonium formate buffer (20 mM, pH = 8.5). The bag was sealed and placed in a test tube with 6 mL of ethyl acetate and sonicated for 15 min. Then, the bag was removed and the extracted solvent was evaporated under nitrogen stream at room temperature. The remaining residue was dissolved in 200 µL of the mobile phase and placed in an autosampler vial prior to analysis.

## Figures and Tables

**Table 1:** Parameters of the MS/MS and ion source conditions.

Analyte	Precursor Ion [M + H]^+^ [*m*/*z*]	Product Ions [*m*/*z*]	Collision Energy [V]	Q1 Prerod [V]	Q3 Prerod [V]
NNK	208.00	79.10 ^1^	−26	−40	−29
		148.20	−11	−12	−13
NNN	178.10	120.10 ^1^	−14	−19	−25
		148.20	−21	−13	−30
NAT	190.00	79.10 ^1^	−14	−29	−13
		160.20	−14	−11	−16
NAB	192.10	162.20 ^1^	−10	−14	−29
		133.20	−14	−19	−29
NNK-d4	212.20	126.20 ^1^	−10	−12	−22
		152.15	−10	−13	−30
NNN-d4	182.20	152.20 ^1^	−14	−12	−14
		122.10	−12	−29	−12
NAT-d4	194.20	164.20 ^1^	−20	−12	−16
		110.20	−10	−17	−23
NAB-d4	196.20	166.20 ^1^	−14	−13	−30
		137.20	−19	−23	−23

^1^ quantification ion.

**Table 2:** Data gathered from the equations of the calibration curves.

Analyte	Calibration Curve Equation (6 Points, *n* = 3)	S_a_	S_b_	LOD [ng/g]	LOQ [ng/g]	R^2^
NNK	y = 0.01211x + 0.0045	0.00029	0.0031	0.9	2.6	0.9931
NNN	y = 0.08552x − 0.0014	0.00070	0.0075	0.3	0.9	0.9992
NAT	y = 0.05133x − 0.0016	0.00041	0.0044	0.3	0.9	0.9992
NAB	y = 0.05865x + 0.00022	0.00073	0.00791	0.4	1.3	0.9981

**Table 3:** Recoveries, SDs, CVs and repeatability—data gathered from spiked samples.

Analyte	Spiking Level	Mean Recovery [ng/g] (%), *n* = 6	SD [ng/g]	CV [%]	Repeatability, *n* = 6			
					Day	Mean Recovery [ng/g] (%)	SD [ng/g]	CV [%]
NNK	5	4.7 (93)	0.2	3.3	1	19.0 (95)	0.7	3.6
	20	19.0 (95)	0.7	3.6	2	19.3 (96)	0.6	2.9
	40	41.5 (104)	0.9	2.1	3	18.5 (92)	0.8	4.2
NNN	5	4.9 (97)	0.2	4.0	1	20.9 (105)	0.8	3.7
	20	20.9 (105)	0.8	3.7	2	21.2 (106)	0.9	4.1
	40	41.4 (104)	0.9	2.2	3	20.5 (103)	0.7	3.2
NAT	5	4.6 (92)	0.2	3.2	1	18.0 (90)	0.5	2.5
	20	18.0 (90)	0.5	2.5	2	17.7 (88)	0.3	1.8
	40	34.8 (87)	0.7	1.9	3	18.6 (93)	0.6	3.2
NAB	5	4.9 (97)	0.2	4.9	1	19.4 (97)	0.4	2.2
	20	19.4 (97)	0.4	2.2	2	18.9 (95)	0.8	4.1
	40	37.5 (94)	0.7	1.9	3	18.7 (93)	0.6	3.3

**Table 4:** Determined concentrations of tobacco-specific nitrosamines (TSNAs) in replacement liquids—Analysis of real samples.

No. Sample and Flavour	Amount of Sample [g]	PG/VG Ratio; (Density) [g/cm^3^]	NNK ± SD [ng/mL]	NNN ± SD [ng/mL]	NAT ± SD [ng/mL]	NAB ± SD [ng/mL]
**Producer 1**		50/50; (1.15)				
1. Cherry	1.059		1.20 ± 0.05	-	-	-
**Producer 2**		60/40; (1.13)				
2. Fruit mix	1.102		2.6 ± 0.3	7.5 ± 0.2	-	-
3. Tobacco	1.092		7.6 ± 0.4	-	1.7 ± 0.1	-
4. Vanilla	1.088		7.5 ± 0.6	4.1 ± 0.6	-	-
5. Mint	1.095		2.9 ± 0.3	15.6 ± 1.3	-	-
**Producer 3**		60/40; (1.13)				
6. Apple	1.066		54.3 ± 3.0	4.2 ± 0.2	-	-
7. Mint	1.057		-	4.6 ± 0.2	-	-
**Producer 4**		80/20; (1.08)				
8. Strawberry	1.047		16.1 ± 0.9	-	-	-
9. Tobacco	1.031		35.8 ± 2.0	30.2 ± 1.1	2.5 ± 0.1	-

## Supplementary Materials

The following are available online at https://www.mdpi.com/1420-3049/24/24/4618/s1, Figure S1: The influence of acetone (ACTN) on the peak shape and resolution between NAT and NAB, Figure S2: Chromatograms of (a) sample 6 and (b) sample 9 obtained after the dilution (100×) with mobile phase.
