# Supplementary material for: Ultrasound-Assisted Solvent Extraction of a Porous Membrane Packed Sample for the Determination of Tobacco-Specific Nitrosamines in the Replacement Liquids for E-Cigarettes
Source: Molecules. 2019 Dec 17;24(24):4618. doi: 10.3390/molecules24244618 (PMC6943691; doi:10.3390/molecules24244618)
Supplement: Supplementary file 1 [file molecules-24-04618-s001.pdf]

Article

Ultrasound-assisted solvent extraction of a porous membrane packed sample for the determination of tobacco-specific nitrosamines in the replacement liquids for e-cigarettes

Paweł Kubica*

Department of Analytical Chemistry, Faculty of Chemistry, Gdansk University of Technology, 11/12 Narutowicza Str., Gdańsk 80-233, Poland, pawkubic@pg.edu.pl

* Correspondence: pawel.kubica@pg.edu.pl

P. Kubica: ORCID number 0000-0003-0576-9495

Abstract: The content of tobacco-specific nitrosamines (TSNAs) possessing carcinogenic properties has been an important area of research since replacement liquids were introduced for e-cigarettes. A method for determining *N′*-nitrosonornicotine (NNN), 4-(methylnitrosamino)-1-(3-pyridyl)-1-butanone (NNK), *N′*-nitrosoanatabine (NAT), and *N′*-nitrosoanabasine (NAB) in replacement liquids for electronic cigarettes was developed using liquid chromatography–tandem mass spectrometry with electrospray ionisation (HPLC-ESI-MS/MS) in the multiple reaction monitoring mode. The sample preparation of replacement liquids was accomplished via the ultrasound-assisted solvent extraction of a porous membrane packed sample. The sample preparation proved to be successful in extracting the analytes, with recoveries from 87% to 105%, with coefficients of variation < 4.9%. Moreover, the linearity and limits of detection and quantitation (LOD, LOQ), together with repeatability and accuracy, were determined for the developed method. The proposed sample preparation and developed chromatographic method were successfully applied to the determination of TSNAs in 9 replacement liquid samples. The NNK and NNN were found to be most frequently detected (89 and 67%, respectively), with concentration ranges from 1.2–54.3 ng/mL and 4.1–30.2 ng/mL, respectively, while NAT was detected with frequency of 22% with range 1.7–2.5 ng/mL and NAB were found to be below the LOD in all samples.

Keywords: e-cigarettes; replacement liquids; tobacco-specific nitrosamines; porous membrane; liquid chromatography–tandem mass spectrometry

SUPPLEMENTARY MATERIAL

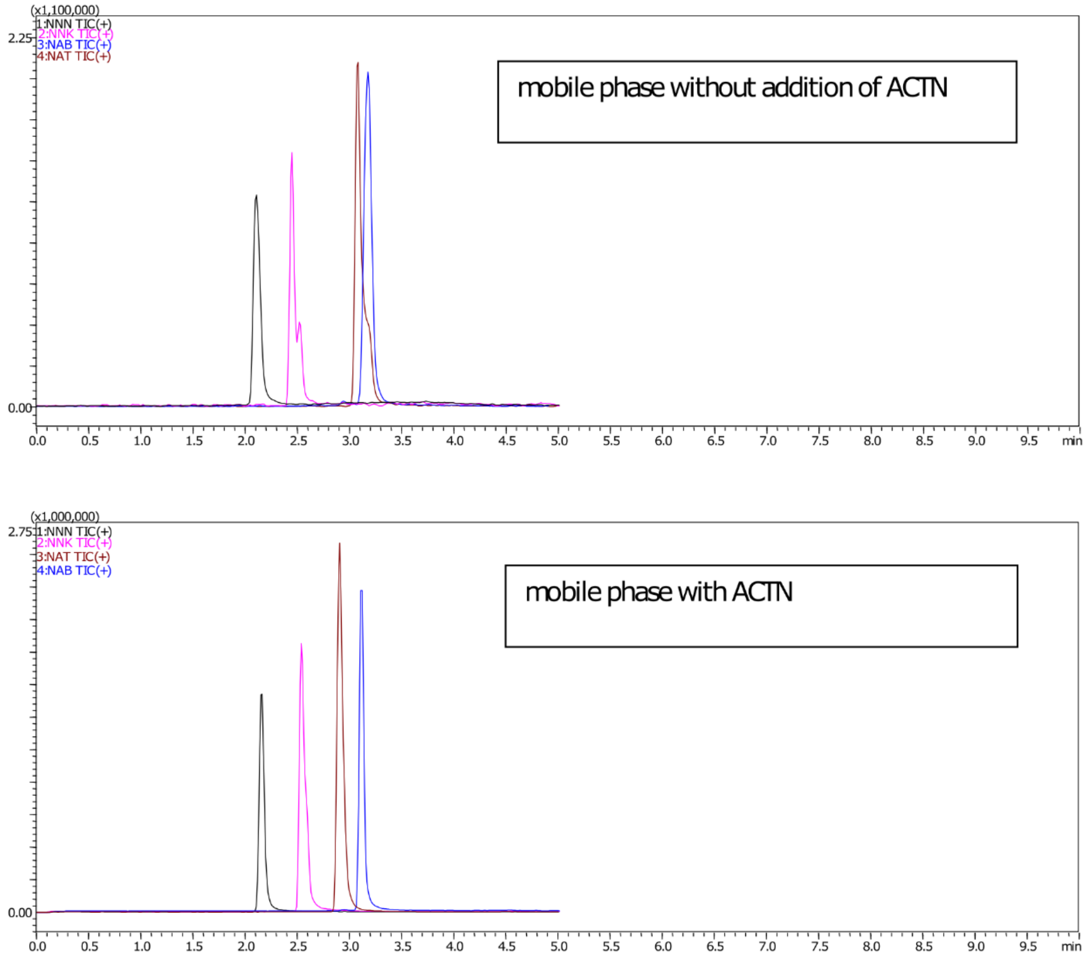

Figure S1. The influence of acetone (ACTN) on the peak shape and resolution between NAT and NAB

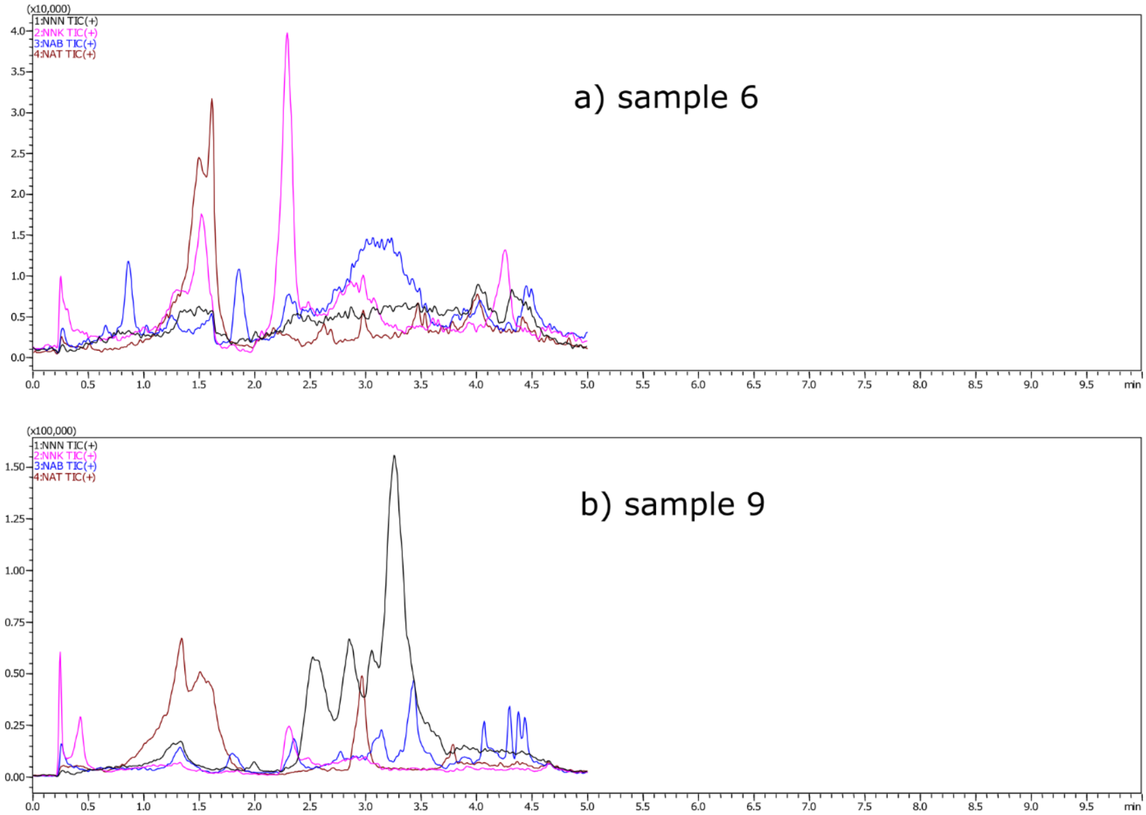

Figure S2. Chromatograms of a) sample 6 and b) sample 9 obtained after the dilution (100x) with mobile phase.
